# Activation of Intracellular Calcium by Multiple Wnt Ligands and Translocation of β-Catenin into the Nucleus: A CONVERGENT MODEL OF Wnt/Ca^2+^ AND Wnt/β-CATENIN PATHWAYS*[Fn FN2]

**DOI:** 10.1074/jbc.M112.437913

**Published:** 2013-10-24

**Authors:** Christopher Thrasivoulou, Michael Millar, Aamir Ahmed

**Affiliations:** From the ‡ Research Department of Cell and Developmental Biology, The Centre for Cell and Molecular Dynamics, Rockefeller Building, University Street, University College London, London WC1E 6JJ, United Kingdom,; the § MRC Centre for Reproductive Health, Queens Medical Research Institute, University of Edinburgh, 47 Little France Crescent, Edinburgh EH16 4TJ, United Kingdom, and; the ¶ Prostate Cancer Research Centre, Division of Surgery, 3rd Floor Laboratories, Charles Bell House, 67 Riding House Street, University College London, London W1W 7EJ, United Kingdom

**Keywords:** Beta-Catenin, Calcium Signaling, Cell Signaling, Nuclear Membrane, Wnt Signaling, Canonical and Non-canonical Signaling, Intranuclear Calcium, Macromolecular Transport, Nuclear Membrane Potential

## Abstract

Ca^2+^ and β-catenin, a 92-kDa negatively charged transcription factor, transduce Wnt signaling via the non-canonical, Wnt/Ca^2+^ and canonical, Wnt/β-catenin pathways independently. The nuclear envelope is a barrier to large protein entry, and this process is regulated by intracellular calcium [Ca^2+^]*_i_* and trans-nuclear potential. How β-catenin traverses the nuclear envelope is not well known. We hypothesized that Wnt/Ca^2+^ and Wnt/β-catenin pathways act in a coordinated manner and that [Ca^2+^]*_i_* release facilitates β-catenin entry into the nucleus in mammalian cells. In a live assay using calcium dyes in PC3 prostate cancer cells, six Wnt peptides (3A, 4, 5A, 7A, 9B, and 10B) mobilized [Ca^2+^]*_i_* but Wnt11 did not. Based upon dwell time (range = 15–30 s) of the calcium waveform, these Wnts could be classified into three classes: short, 3A and 5A; long, 7A and 10B; and very long, 4 and 9B. Wnt-activated [Ca^2+^]*_i_* release was followed by an increase in intranuclear calcium and the depolarization of both the cell and nuclear membranes, determined by using FM4-64. In cells treated with Wnts 5A, 9B, and 10B, paradigm substrates for each Wnt class, increased [Ca^2+^]*_i_* was followed by β-catenin translocation into the nucleus in PC3, MCF7, and 253J, prostate, breast, and bladder cancer cell lines; both the increase in Wnt 5A, 9B, and 10B induced [Ca^2+^]*_i_* release and β-catenin translocation are suppressed by thapsigargin in PC3 cell line. We propose a convergent model of Wnt signaling network where Ca^2+^ and β-catenin pathways may act in a coordinated, interdependent, rather than independent, manner.

## Teaser

**Background:** Cellular transducers of Wnt signaling, β-catenin and Ca^2+^, are considered to act via independent canonical and non-canonical pathways.

**Results:** Six Wnt ligands activated cytosolic and intranuclear Ca^2+^ and nuclear translocation of β-catenin.

**Conclusion:** Wnt β-catenin and Ca^2+^ signaling are coupled in mammalian cells.

**Significance:** A coupled model of Wnt/Ca^2+^ and β-catenin signaling that challenges the canonical and non-canonical categorization is proposed.

## Introduction

Wnt (1) binding to its plasma membrane receptors (Frizzled or FZDs (2)) can activate multiple intracellular transducers (3–6), including the two major transducers β-catenin and Ca^2+^ via the canonical or non-canonical signaling pathways, respectively (7). Activation of gene transcription via canonical, Wnt/β-catenin signaling is the most studied Wnt pathway (7) and is known to play a key role in development and disease (8). Canonical signaling pathway leads to desequesterization of β-catenin from a multiprotein complex (9) followed by activation of transcription of LEF/TCF responsive genes. However, events downstream of receptor activation are dependent upon translocation of desequestered cytosolic β-catenin, a negatively charged 92-kDa protein, traversing the nuclear envelope (NE)^2^ to enter the nucleoplasm (10). Activation of the non-canonical, β-catenin-independent, Wnt/Ca^2+^pathway results in the mobilization of free intracellular calcium [Ca^2+^]*_i_* (11) that regulates many cellular processes (12, 13), including cytoskeleton and cell motility of prostate cancer cells (14). The so-called canonical and the non-canonical pathways are considered to operate independently (15) or antagonistically (16–18). Such binary distinctions of Wnt signaling have come under scrutiny recently (19) (20), but this interpretation has been challenged rarely and only for one ligand of the 19 Wnt ligands, namely, Wnt5A (21, 22).

The NE acts as a barrier that limits ion and macromolecular exchange into the nucleoplasm. NE consists of an inner nuclear membrane facing the nuclear lamina, separated by a cisterna from the outer membrane that is contiguous with the endoplasmic reticulum. Invaginations of the supramolecular nuclear pore complex (NPC) provide the only route for the exchange of matter (with an upper limit of ∼90–100 kDa) between the cell cytoplasm and nucleoplasm (23). Macromolecular transport across the NE is a complex process requiring protein recognition by specific amino acid motifs (*e.g.* nuclear localization signal sequence or the Armadillo domain repeat sequences) on the imported protein, first binding to the outer membrane of the NE and then traversing through the NPC by an energy expenditure mechanism such as GTPase Ran/transport receptor pathways (24). The 92-kDa β-catenin, unlike small macromolecules (<10 kDa, (25)) is at the upper limit for macromolecule entry through the NPC into the nucleoplasm and does not involve simple diffusion (9) or the Ran GTPase pathway (26–28), suggesting that β-catenin may encounter a considerable hurdle to traverse the NE.

The NE is also an ion-selective membrane with a dynamic trans-NE electrical potential that is responsive to changes in Ca^2+^ and K^+^ concentrations (reviewed by Mazzanti *et al.* (23)). Cytosolic [Ca^2+^]*_i_* regulates nuclear Ca^2+^ concentration (29); an increase in nucleoplasmic Ca^2+^ facilitates transport of large macromolecules (>10 kDa) across the NE (25, 30, 31), whereas a decrease in nucloeplasmic Ca^2+^ inhibits trans-NE macromolecular transport (25, 32–34). The cell nucleus has been termed a “negatively charged sink” (23) consisting largely of negatively charged DNA and regions of nucleosome core proteins (35). In HeLa cells, the trans-NE potential has been measured at −43 mV with an estimated electrical pore diameter of 11 μm^−2^ (23, 36). In addition to its bulk, the β-catenin protein has an overall negative charge (−20.8 at pH 7.0). Thus, the NE presents a physical as well as an electrical barrier for β-catenin entry into the nucleoplasm, indicating a physicoelectrochemical mechanism for β-catenin translocation across the NE.

We hypothesized that a Wnt-mediated increase in [Ca^2+^]*_i_* leads to an increase in the nucleoplasmic calcium and depolarization of NE that facilitates the passage of β-catenin across the NE. Identity of Wnts that can activate [Ca^2+^]*_i_* release in mammalian cells is not well characterized. In prostate cancer cells, Wnt5A induces a large increase in [Ca^2+^]*_i_* (14), but it is not known whether this causes an increased nucleoplasmic Ca^2+^ and depolarization of the NE, and if there is subsequent translocation of β-catenin to the nucleoplasm. To test our hypothesis, we first determined (i) whether Wnts other than Wnt5A also increase in [Ca^2+^]*_i_*; (ii) that increase in [Ca^2+^]*_i_* due to Wnt/Ca^2+^ signaling activation results in depolarization of nuclear membranes; (iii) that in cells, activated by multiple Wnt ligands, β-catenin is translocated into the nucleus. By using a live fluorochrome assay and subsequent assessment of β-catenin translocation to nucleoplasm in Wnt ligand-activated mammalian cancer cell lines (PC3, prostate, MCF7, breast, and 253J bladder cell lines), we demonstrate that a number of Wnt ligands increase intracellular and intranuclear Ca^2+^, with distinct kinetics, depolarization of the NE and translocation of β-catenin to the nucleus; both the increase in Wnt induced [Ca^2+^]*_i_* release and β-catenin translocation are suppressed by thapsigargin, an inhibitor of microsomal Ca^2+^-ATPase (37). A model in which Wnt/Ca^2+^ and Wnt/β-catenin pathways are coupled rather than independent, as thought previously, is proposed.

## EXPERIMENTAL PROCEDURES

### 

#### 

##### Cell Lines

All cell lines (PC3, prostate cancer, MCF7, breast cancer, 253J bladder cancer, and Cos 7 simian kidney) were obtained from ATCC via John R. W. Masters, University College London and cultured in RPMI 1640 (Invitrogen) medium containing 5 mm l-glutamine and FBS.

##### Wnt Peptides

Recombinant Wnt (3A, 4, 5A, 7A, 9B, 10B, and 11) peptides were purchased from R&D Systems (Minneapolis, MN). Wnt-peptide stock solutions were prepared in PBS.

##### [Ca^2+^]_i_ and Membrane Potential Imaging

The imaging protocol was developed in our laboratory and has been described in detail elsewhere (14). Briefly, 100000-200000 PC3, MCF7, 253J, or Cos 7 cells were plated on 35-mm FluoroDish (World Precision Instruments) and grown for 48 h; calcium indicators Fluo-4 (Invitrogen) and FuraRed (Invitrogen) at 1 ng/ml were added to FluoroDish containing only 1 ml of culture medium and incubated for 15–20 min at 37 °C. The culture medium was removed and washed (2×) and replaced with 1 ml of PBS (GIBCO). Live cell calcium imaging was performed on an Olympus FluoView FV 1000 confocal microscope (Olympus KeyMed) equipped with a 20× dry objective (numerical aperture = 0.75). Calcium indicators were excited with an argon laser line (488 nm), and emissions were recorded in the green channel (500–560 nm) for Fluo-4 and red channel (600–700 nm) for FuraRed after addition of vehicle control (PBS) or Wnt X (30–600 ng/ml). Images were acquired every 1.1 or 2.2 s for up to 600–800s; image and data acquisition was performed using FluoView 1000 software (Olympus). Fluorescent intensity was measured and data exported as a tab delimited file for further analysis using a spreadsheet. All imaging experiments were performed in duplicate; intracellular calcium was measured in at least 3–12 different passages (passage numbers 25 to 37) of cell line for each Wnt ligand at 100 ng/ml. Wnt 11 was tested in at least five different passages at three different concentrations. To test whether the Wnt-activated calcium release was from [Ca^2+^]*_i_* stores, in some cases PC3 cells, loaded with calcium dyes, were incubated with 1–5 μm of thapsigargin (Sigma), resuspended in dimethyl sulfoxide, for 20 min at 37 °C, prior to addition of Wnt ligand and live calcium imaging (as described above).

Experiments with the membrane potential sensitive dye FM4 64 (Invitrogen) and Fluo4 were performed in exactly the same manner as for the Fluo4/FuraRed but with different emission spectra detection for the FM4 64 indicator, *i.e.* excitation with 488 nm for both indicators and emission detection at 500–560 nm for Fluo4 and 650–800 for FM4-64. All other imaging/data acquisition conditions were the same as above.

The experiments were performed using a 35-mm, glass-bottomed FluoroDish containing 1 ml of PBS at 37 °C to which ligands were added at desired concentration. In a static, non-superfusing assay system, there is a lag time before the start of a response, in this case, an increase in the intensity of the Fluo4 signal due to the release of free calcium. To measure the diffusion constant under these conditions, we used pontamine sky blue (Sigma), which fluoresces in the red spectrum with an excitation of 488 nm, using the standard practice for adding ligands; the diffusion constant was measured to be 23.3 ± 2.1 μm·s^−1^ (means ± S.E., *n* = 8). The calcium wave (supplemental Movies), in response to Wnt ligands, traveled across the FluoroDish.

##### Data Analysis

After image acquisition, regions of interest were drawn over individual PC3 cells (up to 25) in a single frame, and calcium intensity measurements were made using FluoView 1000 software (Olympus). The measurements were exported as a tab-delimited file into a spreadsheet for further analysis, and Fluo4/Fura Red ratios were calculated. The waveform, a product of the ratio of Fluo4 and FuraRed over the period of measurement was used to measure the kinetics of the activation of [Ca^2+^]*_i_* release in PC3 cells in response to different Wnt ligands. The time constants (rise, dwell, and fall times) were calculated by first fitting the Fluo4/FuraRed ratio to a single Gaussian function (OriginLab Corp.); a lower (10%) and upper (90%) amplitude thresholding method was then used to calculate the time constants (s). Statistical significance was calculated using Mann-Whitney U test.

##### Immunocytochemistry of Mammalian Cell Lines

Immunostaining was performed in two different laboratories, independently, with the experimenter blind to the identity of the treatment. Either cells used in FluoroDish, post [Ca^2+^]*_i_* experiments using dyes (see above), or grown in eight-well chamber glass slides (Lab Tek II, Nunc) were used. For Wnt ligand treatment, the culture medium replaced with PBS (without calcium) at 37 °C. The experiments were replicated for Wnt ligand or vehicle control as described for [Ca^2+^]*_i_* measurements. Briefly, Wnt ligands were added and chamber slides incubated at 37 °C for up to 20 min. Chamber wells were washed 3× with 0.5 ml of PBS, and cells were fixed with 0.5 ml of 4% formalin for 30 min at 4 °C. Cells were washed 3× with ice-cold PBS and stored in 0.5 ml of PBS at 4 °C for immunostaining. In some experiments, PC3 cells were treated with thapsigargin as described above.

For immunostaining, cells were washed twice for 5 min in TBST (20 mm Tris, pH 7.4, 0.9% NaCl, 0.1% Tween 20; Sigma). Cells were then incubated in 20% normal goat serum/TBST for 30 min at room temperature, the chambers were drained, and cells were incubated with mouse anti-β-catenin (ab22656, Abcam) at 1:500 dilution in normal goat serum/TBST overnight at 4 °C. Following two further 5-min washes in TBST, sections were incubated in goat anti-mouse peroxidase Fab (Abcam) at 1:500 dilution for 30 min at room temperature. Following a further two 5-min washes in TBST, cells were incubated with tyramide Cy3 (PerkinElmer Life Science) for 10 min at room temperature. Sections were washed twice for 5 min in TBST before nuclear counterstaining with DAPI. After a final two 5-min washes, the chambers were removed, and a coverslip was placed on slides using Permafluor (Thermo Shandon).

##### Imaging and Quantitation for β-Catenin Expression and Nuclear Co-localization Analysis

Fluorescence immunocytochemical staining for β-catenin expression in cell lines was imaged using Olympus Fluoview 1000 confocal system (Olympus KeyMed) mounted on an Olympus IX81 inverted microscope using a 20 × 0.7 numerical aperture or 40 × 1.0 numerical aperture dry objective with a zoom of 3×. DAPI (nuclear counterstain) was excited at 405 nm with an emission of 415–500 nm; Cy3 (β-catenin-labeled) signal was excited at 561 nm with an emission spectrum between 570–670 nm. Laser power, gain, and off-set settings for the photomultipler tubes were kept similar for comparable samples. Z-stacks of at least three fields per sample were imaged and saved in Olympus FV (OIF or OIB) file format.

Nuclear co-localization of β-catenin was calculated by importing FV files for untreated, control, or treated cells, into colocalization plug-in of Fiji software (38) for Cy3 (β-catenin) and DAPI (nuclear) signals. Pearson co-relation of colocalization were tabulated for 25–90 cells (from two to five different passages of cells) using regions of interest analysis. Statistical significance between untreated and treated cells was calculated using a Mann-Whitney U test.

##### Western Blotting

Cell membrane fraction from control untreated and Wnt-treated PC3 and MCF7cells was isolated by differential centrifugation. Protein was quantified using a BCA protein assay and 10 μg/lane was loaded onto a 10% SDS-PAGE gel. Samples were treated with protease and phosphatase inhibitors (Roche Applied Science). The resolved protein was transferred onto a PVDF membrane and that was cut into strips of 250 to 75 and 70–35 kDa as visualized by using Rainbow molecular weight markers (Invitrogen). The two PVDF membrane strips were probed with the following antibodies: anti-phospho-LRP-6 (Ser-1490) that detects human LRP-6 when phosphorylated at Ser-1490 in Western blots (200 kDa, R&D Systems, AF6649, 2.5 μg/ml) and β-actin (42 kDa, Abcam, ab6276, 1:5000 (diluted)). Following incubation with appropriate secondary antibodies and ECL detection, the blots were exposed on Kodak BioMax light film (Carestream Health) in an autoradiography cassette.

##### Scratch Wound Assay

PC3 cells were grown in 24-well ImageLock plates (Essen Bioscience) to confluency and scratched using a wound maker device (Essen Bioscience). Imaging (at 2h intervals) and quantitation of wound width was performed as described previously (14) using Incucyte (Essen Bioscience). The rate of wound closure (± Wnts and thapsigargin) was calculated by fitting the line using linear regression function in Origin software (OriginLab, Corp.). Statistical significance of difference between the slopes for untreated and treated samples was calculated using F-test.

## RESULTS

We have shown previously that Wnt/Ca^2+^ signaling is a key regulator of cytoskeleton and motility in PC3 cells with a 2.5-fold increase in [Ca^2+^]*_i_* (14). Little is known about the ability of Wnts to induce [Ca^2+^]*_i_* release in mammalian cells, except that Wnt5A induces intracellular Ca^2+^ release in prostate cells. The first question we sought to answer was which other Wnts induce [Ca^2+^]*_i_* release in PC3 cell lines?

### 

#### 

##### [Ca^2+^]_i_ Release in Response to Wnt Ligands

**FIGURE 1.**
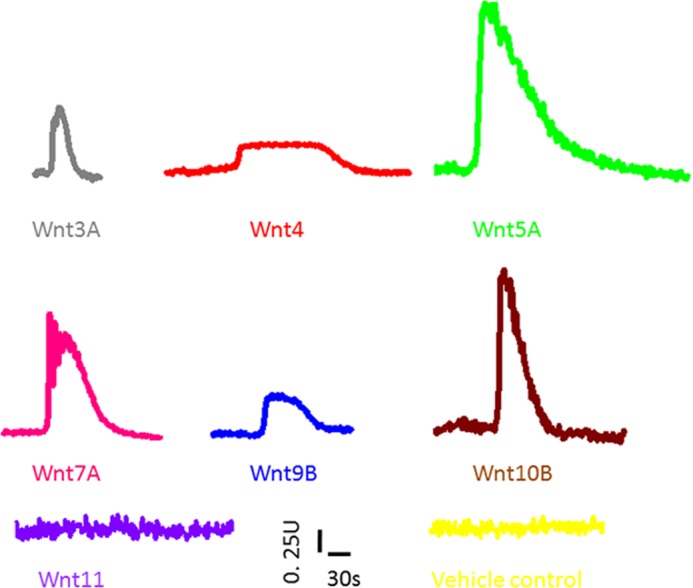
**Representative trace of intracellular calcium release in PC3 cells in response to addition of 100 ng/ml Wnt ligand peptide (*gray*, *Wnt3A*; *red*, *Wnt4*; *green*, *Wnt5A*; *pink*, *Wnt7A*; *blue*, *Wnt9B*; *brown*, *Wnt10B*; *violet*, *Wnt11*; *yellow*, *vehicle control*).** All of the Wnts tested, except for Wnt11, induce intracellular calcium mobilization in PC3 prostate cancer cell line. The *x* axis is time (s), and the *y* axis is the ratio of Fluo4/Fura Red (see the supplemental Movie for each ligand). The waveform for each ligand was used to calculate time constants (rise, dwell, and fall time) of calcium release.

Addition of all Wnt ligands tested, with the exception of Wnt11, caused a change in ratio of Fluo4/Fura Red in PC 3 cells (supplemental Movies SM1–SM8). The ratio of Fluo4/FuraRed was plotted as a function of time (Fig. 1, representative trace for all Wnts tested), and the waveform was used to measure the kinetics of the activation of [Ca^2+^]*_i_* in PC3 cells in response to different Wnt ligands. Parameters such as the amplitude and time constants (rise, dwell, and fall times) were calculated by first fitting the Fluo4/FuraRed ratio to a single Gaussian function. We then used a lower (10%) and upper (90%) amplitude thresholding method to calculate the timing parameters. The amplitude for [Ca^2+^]*_i_* release in response to Wnt ligands represents the peak of the calcium waveform. The amplitude of calcium release was Wnt ligand concentration-dependent (supplemental Fig. S1). A number of Wnt ligands (*e.g.* 5A, 9B, and 10B) also activated [Ca^2+^]*_i_* release in MCF7 and 253J human breast and urinary tract cell lines, respectively (supplemental Movies SM9 – SM12), indicating that this phenomenon was not specific to PC3 cell line. Furthermore, Wnt5A also activated [Ca^2+^]*_i_* in Cos7 simian kidney cell line (supplemental Movie SM13).

**FIGURE 2.**
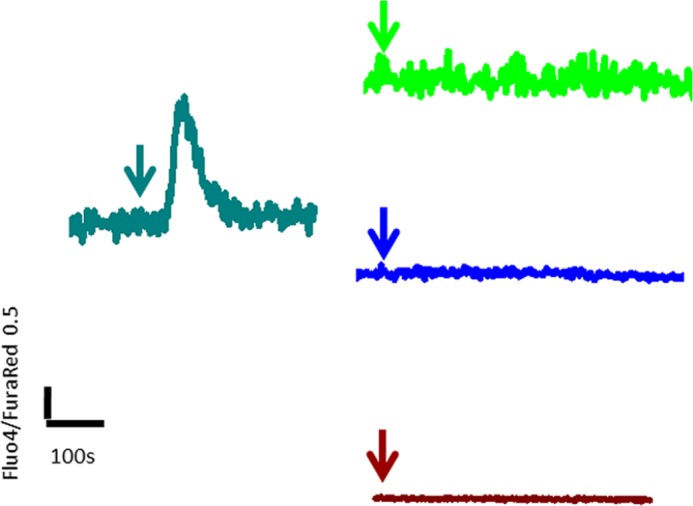
**[Ca^2+^]*_i_* measurements were made as described under “Experimental Procedures” using Fluo4 and FuraRed dyes.** Thapsigargin (at 5 μm final concentration) was added at the time indicated (*arrow*). A representative trace of a calcium transient (*cyan*) evoked after the addition of thapsigargin is shown. The calcium signal (ratio of Fluo4/FuraRed) declined after an initial rise to reach the baseline within 120 s. In PC3 cells incubated with thapsigargin (5 μm) for 15 min, addition of Wnt5A (100 ng/ml, *green*), Wnt9B (200 ng/ml, *blue*), or Wnt 10B (200 ng/ml, *brown*) did not evoke an increase in [Ca^2+^]*_i_* as seen in untreated PC3 cells (Fig. 1).

Thapsigargin is an inhibitor of the endoplasmic reticulum Ca^2+^-ATPase and depletes [Ca^2+^]*_i_* stores (37). Thapsigargin has been used previously to investigate ligand-induced [Ca^2+^]*_i_* release (39). The increase in [Ca^2+^]*_i_* in response to Wnt ligands tested (5A, 9B, and 10B) was abolished after treatment of PC3 cells with thapsigargin (5 μm) (Fig. 2). These results indicate that depletion of [Ca^2+^]*_i_* abolishes Wnt induced [Ca^2+^]*_i_* release.

##### Rise, Dwell, and Fall Time of [Ca^2+^]_i_ Release

**FIGURE 3.**
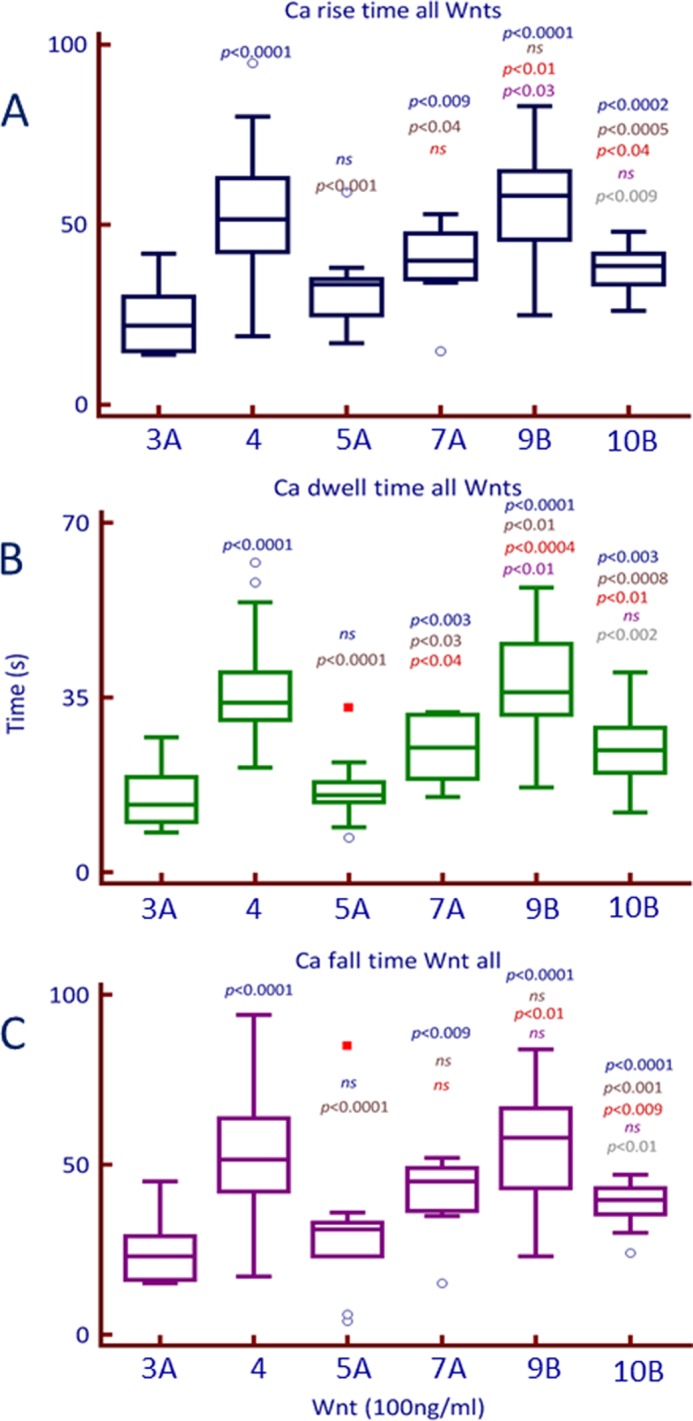
**Box plot for time constants of the Wnt-induced calcium waveform.** The time constants (*A*, rise; *B*, dwell; and *C*, fall times) were calculated from calcium waveform generated by the addition of each Wnt ligand (100 ng/ml) to PC3 cells. Significance of difference was calculated using a Mann-Whitney U test. All time constants were compared with Wnt3A, primarily; subsequent comparisons (*e.g.* Wnt4 to Wnt5A, Wnt4 to Wnt7A etc) are listed in descending order on *top* of box plots for each subsequent Wnt. *ns*, difference was not statistically significant.

Time constants for the rise, dwell, and fall time of [Ca^2+^]*_i_* release in response to Wnt peptides (100 ng/ml) were calculated as described under “Experimental Procedures” and shown in Fig. 3. Wnt3A and Wnt5A showed the shortest rise and fall time, whereas Wnt 9B and Wnt4 showed the slowest rise and fall time (Fig. 3, *A* and *C*). DTs reflect the time the [Ca^2+^]*_i_* signal was sustained at peak amplitude after application of Wnt peptides. The DT analysis (Fig. 3*B*) indicates that of the six Wnts activating [Ca^2+^]*_i_* release could be classified into three classes: short, 3A and 5A (DT < 15 s); long, 7A and 10B (DT > 25 s); and very long, 4 and 9B (DT > 30 s).

##### Wnt Ligands Activate Calcium by a Negative Cooperative Mechanism

**FIGURE 4.**
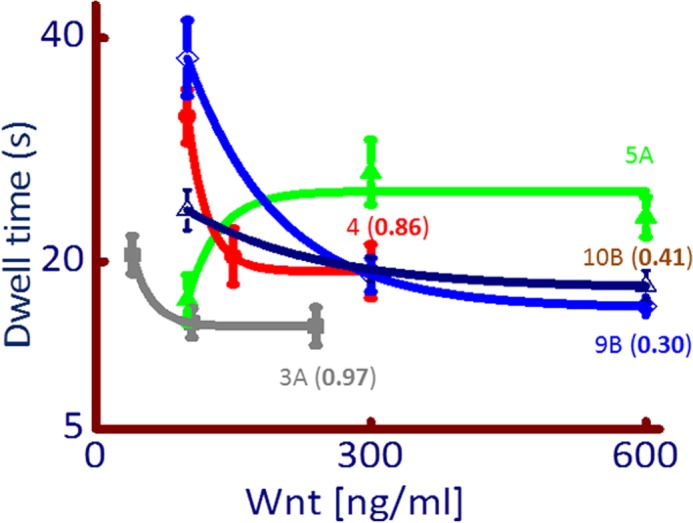
**Wnts that activate [Ca^2+^]*_i_* release show negative cooperativity.** Wnts 3A (*gray*), 4 (*red*), 9B (*blue*), and 10B (*dark blue*) showed decreasing and significant difference between at least two incremental increasing concentrations for the dwell time for [Ca^2+^]*_i_* release. The dwell time constant at increasing Wnt concentration are fitted to a first-order reaction equation (*y*0+A1e (−*x*/t1)), where *y*0 is Y offset, A1 is amplitude; and t1 is decay constant. The rate constant for the reaction (*k*) is given in brackets for each Wnt ligand. No significant difference was found in the dwell times for different concentration for Wnt7A, and therefore, the data is not considered for this analysis. Wnt5A (*green*) showed a classic Michaelis-Menten kinetics with an estimated *K*½ of 67 ng/ml.

The kinetics of [Ca^2+^]*_i_* release was further analyzed by using three different concentrations for each Wnt ligand. Box-whisker plots for the rise, dwell, and fall time as a function of low, medium, and high concentrations of each Wnt ligand is given in supplemental Figs. S2–S7). Time constants were significantly different between at least two different concentrations for Wnts 3A, 4, 5A, 9B, and 10B (supplemental Figs. S2-S4, S6, and S7), but no difference in these time constants was observed for Wnt7A (supplemental Fig. S5). We compared the DT constants for Wnts 3A, 4, 5A, 9B, and 10B in more detail to elucidate the mechanisms of [Ca^2+^]*_i_* release by fitting the DT data to calculate the reaction-rate expression for a first-order reaction (Fig. 4). Strikingly, Wnts 3A, 4, 9B, and 10B show negative cooperativity but not Wnt5A. Negative cooperativity indicates a decrease in function (DT) as the ligand concentration increases; the rate constants for Wnts showing negative cooperativity are given in Fig. 4. Wnt5A was the only [Ca^2+^]*_i_* activating Wnt ligand that follows the classical Michaelis-Menten kinetics, with a half-maximal concentration of 67 ng/ml. These results provide the first evidence of negative cooperativity of binding of Wnts (3A, 4, 9B, and 10B) to their receptor and show, also for the first time, that the molecular dynamics of [Ca^2+^]*_i_* release, via receptor/ligand interaction by different Wnts, could provide critical regulatory mechanisms of Wnt/Ca^2+^ signaling.

##### [Ca^2+^]_i_ Release Results in Increased Intranuclear Ca^2+^ and Altered Electrical Activity in the Nucleus

**FIGURE 5.**
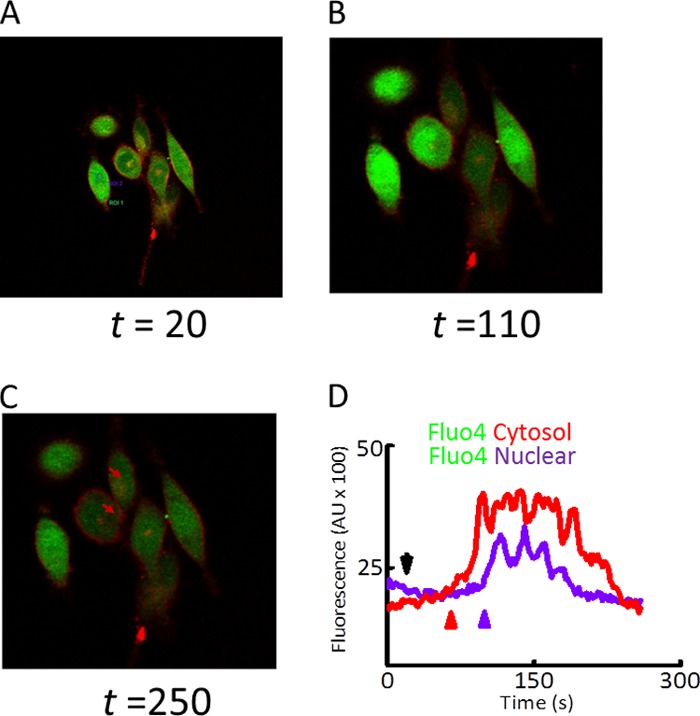
**[Ca^2+^]*_i_* release results in an increase in intranuclear Ca^2+^ concentration.** Snapshots of three time points (*A–C*) at *t* = 20, 110, and 250 s (*A–C*) in PC3 cells loaded with Fluo4 (*green*) and FM4 (*red*) are shown and treated with Wnt5A (100 ng/ml). Quantitative expression of the increase in cytosolic (*red line*, [Ca^2+^]*_i_*) and intranuclear Ca^2+^ (*violet line*) is given in *D*. At *t* = 20 (*A*), steady state, base-line [Ca^2+^]*_i_* as a function of Fluo4 intensity is shown that shows an increase at *t* = 110 s (*B*), subsequent to the addition of Wnt5A (*black arrow*), increase in [Ca^2+^]*_i_* is initiated (*red arrow*) with an increase in intranuclear Ca^2+^ following shortly (∼33 s) later (*violet arrow*). At *t* = 250 s (*C*), there is an increase in the FM4 signal in the cell and nuclear membranes (*brown arrows*). A representative from three independent measurements is shown. Imaging was performed on Leica SP2 confocal and DMRB microscope with 20 × 0.8 numerical aperture water dipping objective and 12-bit gray depth using similar conditions to the FV1000.

Calcium is a key regulator of the electrical activity in the nucleus (23). Intranuclear concentration of Ca^2+^ follows increase in cytosolic Ca^2+^ (29). We therefore investigated changes in intranuclear calcium and membrane potential using cells loaded with Fluo4 and FM4-64 with or without Wnts in PC3 and MCF cells. There was an increase in the [Ca^2+^]*_i_* (Fluo4) signal in the green channel (500–560 nm) upon addition of the Wnt ligands in these cells (supplemental Movies SM14 and SM15). After ∼300 s, there was an increase in the intensity of the FM4-64 signal in the red channel (650–800 nm); there was depolarization of the cell membrane, as expected (Fig. 5). There was also an increase in the intensity of FM4-64 around the nucleus, indicating a depolarization of the transnuclear envelope potential in PC3 cells (Fig. 5; and for MCF7 cells, supplemental Fig. S8).

##### Wnt/Ca^2+^ Signaling and Cell Motility

One functional consequence of inhibition of downstream Wnt/Ca^2+^ signaling is a decrease in cell motility (14). To test whether inhibition of Wnt/Ca^2+^ signaling by Wnt/canonical signaling ligands such as Wnt9B and 10B, we used the wound scratch assay in which Wnt-induced [Ca^2+^]*_i_* release was inhibited by thapsigargin (supplemental Fig. S9). The rate of wound closure in wounded PC3 cells in the presence of Wnt 5A or 9B was similar to that of control untreated cells (supplemental Fig. S9). However, there was a significant decrease in the rate of wound closure in cells treated with thapsigargin even in the presence of Wnt ligands. These results indicate that downstream mechanisms of Wnt/Ca^2+^ signaling are similar for the classical non-canonical (Wnt 5A) or canonical (Wnt 9B) Wnt ligands. Similar results were obtained for Wnts 3A and 10B when [Ca^2+^]*_i_* was inhibited by incubation with thapsigargin (supplemental Table 1).

##### Translocation of β-Catenin to the Nucleus Subsequent to Nuclear Membrane Depolarization

**FIGURE 6.**
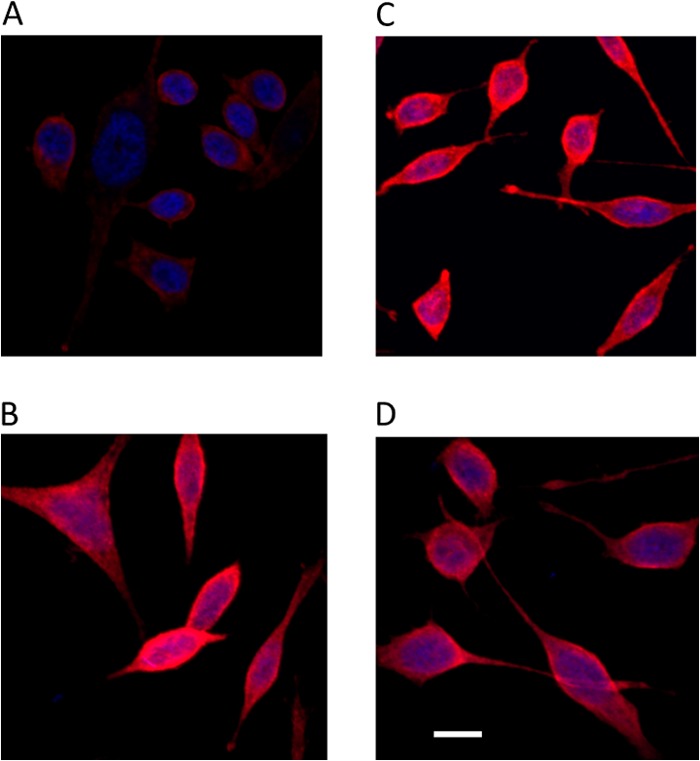
**β-Catenin (*red*) translocation to the nucleus (counterstained with DAPI, *blue*) in response to activation by Wnt ligands (100 ng/ml) in in PC3 prostate cancer cell line, control (*A*) or after activation with Wn5A (*B*), Wnt 9B (*C*), and Wnt 10B (*D*).** Cells were grown in eight-well chamber slides, treated with Wnt ligands, fixed, and stained for β-catenin using protocols described under “Experimental Procedures.” Z-stacks obtained using an Olympus confocal system are shown. Representative images of three individual experiments are shown. *Scale bar*, 10 μm.

**FIGURE 7.**
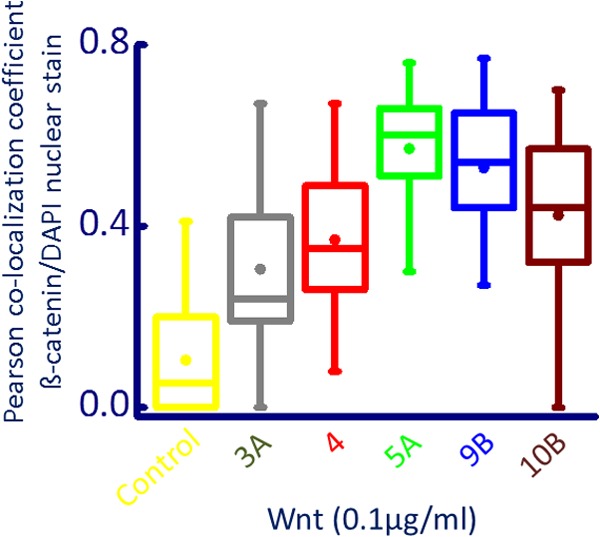
**Box plots of calculated Pearson co-localization coefficient of β-catenin translocation to the nucleus.** Immunohistochemistry and image analysis was performed as described under “Experimental Procedures.” Between 20–97 cells from two to three independent experiments were analyzed. *Circles* represent means of Pearson co-localization coefficient for each condition. Cy3 (label for β-catenin) showed significantly (*p* < 0.001, Mann-Whitney test; see supplemental Table 2) increased co-localization coefficients for Wnt-treated (100 ng/ml) *versus* control cells.

β-Catenin expression was determined subsequent to Wnt ligand activation and [Ca^2+^]*_i_* release using immunofluorescence. Compared with vehicle controls, there was an increased in expression of β-catenin in PC3 cells treated with Wnts5A, 9B, and 10B (Fig. 6). There was little expression of β-catenin in vehicle-treated cells (Fig. 6*A*); the expression of β-catenin is visibly increased in PC3 cells treated with Wnt5A (Fig. 6*B*), Wnt9B (Fig. 6*C*), and Wnt 10B (Fig. 6*D*) and is visible in the nucleus. Similar results were obtained for Wnt 3A and Wnt4 in PC3 cells (supplemental Fig. S10). Co-localization of β-catenin and DAPI signals was quantified using Fiji software (38) as described under “Experimental Procedures.” There was a significant increase in the Pearson coefficient of nuclear co-localization of β-catenin when cells were exposed to Wnts 3A, 4, 5A, 9B, and 10B (Fig. 7 and supplemental Table 2). Furthermore, inhibition of Wnt-induced [Ca^2+^]*_i_* release, by incubation of cells with thapsigargin, reduced the translocation of β-catenin into the nucleus (supplemental Fig. S11), indicating a link between β-catenin translocation, store-operated, thapsigargin-inhibitable [Ca^2+^]*_i_* release and perhaps alterations in nuclear envelope potential.

Wnt ligands are known to phosphorylate their cell surface co-receptors (LRP5/6 (low density lipoprotein receptor-related protein 5/6) (40)), an early event in Wnt signaling activation (41). It appears that both Wnt5A and Wnt9B phosphorylate LRP6 co-receptors in PC3 cells (supplemental Fig. S12).

We also used the paradigm ligands of the Wnt classes (as categorized in Fig. 3) in two other cell lines to determine that an increase in [Ca^2+^]*_i_* release was followed by translocation of β-catenin to the nucleus. The data for MCF7 and 253J cells, with β-catenin expression visible in the cell nucleus after treatment with Wnt ligands, are shown in supplemental Fig. S13. These results indicate that increase in [Ca^2+^]*_i_* in response to a variety of Wnt ligands leads to a depolarization of the nuclear envelope and β-catenin translocation from the cytosol to the nucleus.

## DISCUSSION

Wnt signaling is categorized into independent, canonical, and non-canonical pathways, with β-catenin as a key transducer of the former and Ca^2+^ as a major effector molecule for the latter (other non-canonical pathways include the planar cell polarity (42, 43) and cGMP/phosphodiesterase pathways (44)). The Wnt/β-catenin pathway is used as a synonym for the canonical pathway (45–47). The categorization is not absolute as recent examples show some cross-talk (21, 22); however, the distinction of β-catenin-dependent and -independent pathways to describe Wnt signaling remains unresolved (7). In this report, we have addressed two key questions regarding Wnt signaling in mammalian cells: 1) which and how many Wnt ligands can mobilize intracellular calcium and 2) what are the mechanisms of β-catenin entry into the nucleus? Our data show for the first time that in mammalian cells, six of the seven Wnts tested in a direct calcium measurement assay mobilize [Ca^2+^]*_i_*_,_ including Wnt ligands previously thought to act only via β-catenin pathway (*e.g.* Wnt 9A and 10B) (Fig. 1 and supplemental Movies SM1–SM7). A discharge of [Ca^2+^]*_i_* from the endoplasmic reticulum and inhibition of microsomal Ca^2+^-ATPase by incubation of cells with thapsigargin abolished the increase in Wnt-induced [Ca^2+^]*_i_* release (Fig. 2) and reduced β-catenin translocation to the nucleus (supplemental Fig. S11). Thapsigargin is resuspended in dimethyl sulfoxide. Dimethyl sulfoxide induces Ca^2+^ spikes in a variety of cells (48); our results in PC3 cells concur with previous observations (supplemental Fig. S14). Thapsigargin also induced Ca^2+^ transient, which declined to reach the base line within 120 s (Fig. 2). However, a 15-min incubation with dimethyl sulfoxide had no effect on Wnt-induced increase in [Ca^2+^]*_i_* (supplemental Fig. S14), unlike thapsigargin treatment, which abolished Wnt-induced [Ca^2+^]*_i_* release (Fig. 2), indicating that Wnt activates store-operated Ca^2+^ release.

**FIGURE 8.**
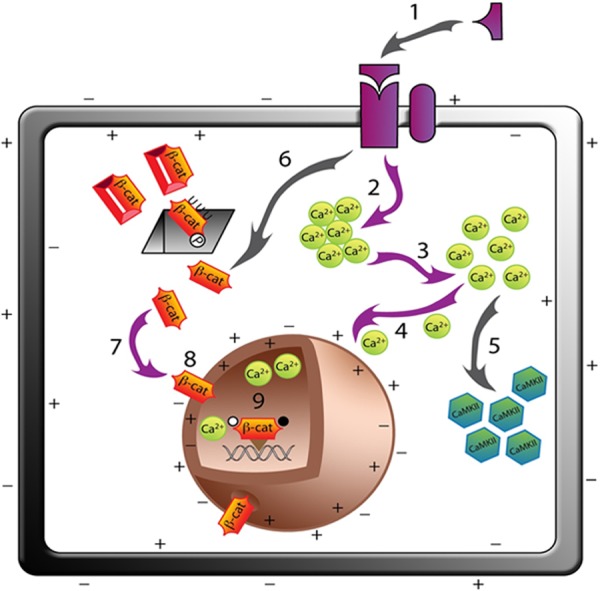
**A convergent model for the canonical (Wnt/β-catenin) and non-canonical (Wnt/Ca^2+^) Wnt signaling, in mammalian cell lines (only the cell membrane and the nucleus shown in the schematic for clarity).** Proposed steps of Wnt signaling in mammalian cells are as follows: binding of Wnts (3A, 4, 5A, 7, 9B, and 10B) (*1*) results in the activation of intracellular calcium stores (*2*) that increase in the intracellular concentration of free store-operated calcium ((*3*) Figs. 1–3 and supplemental Movies). The increase in free calcium depolarizes the cell membrane (supplemental Movies) and as calcium enters the nucleus (*4*), the NE is depolarized (Fig. 5 and supplemental Movies); the calculated diffusion constants for the activation of bound to free intracellular calcium is 0.21 μm/s, and time constants for steps 1 to 4 are likely to be in milliseconds. We have shown, previously, that activation of Wnt/Ca^2+^ pathway by Wnt5A increases (*5*) Ca^2+^/calmodulin-dependent kinase (CamKII) activity to induce cytoskeletal modulation in prostate cancer cells (14). It is well established by previous studies (see Ref. 7 for a review) that activation of Wnt signaling (*6*) desequesters the caged β-catenin (β*-cat*) that is phosphorylated (*P*) and ubiquitinated (*UUU*) prior to degradation in proteasomes, *gray square*) in the cytosol. The desequestered β-catenin (*7*) translocation is facilitated across the NE (Fig. 5), which is now depolarized (*8*) and has increased nucleoplasmic calcium (*9*). β-Catenin is now co-localized in the nucleus (Figs. 6 and 7) where it is known to bind to LEF/TCF proteins to initiate gene transcription. For simplification, many other proteins involved in Wnt signaling are not shown and multiple other pathways such as Wnt/PCP pathways are not included in the illustration. The steps for which evidence is given in the manuscript are shown with *arrows* shaded *purple*. See supplemental Movie SM16 for an animated version of the proposed model.

The results presented here provide first evidence that key transducers of canonical and non-canonical pathways converge to activate Wnt signaling in mammalian cells. A new convergent model of Wnt signaling network is proposed (Fig. 8), in which multiple Wnt ligands in a variety of mammalian cell lines, increase free intracellular and nucleoplasmic Ca^2+^, depolarize the NE, and increase the translocation of β-catenin to the nucleoplasm, suggesting a coupled rather than independent Wnt/Ca^2+^ and β-catenin pathway.

### 

#### 

##### Categorization of Wnt Ligands and Distinction into Canonical and Non-canonical Pathways

The classification of Wnt ligands mediated signaling via the β-catenin or calcium pathways is largely based upon their ability to induce a secondary axis in *Xenopus* embryos (49) or their ability to transform C57 mg cells (50). Ecotopic expression of Wnts such as Wnt1, 3A, 8, and 8B was found to induce a secondary axis in *Xenopus* embryo and stabilize β-catenin and other Wnts such as Wnt5A and 11 did not (12). Members of the Wnt1 class include Wnt1, Wnt2, Wnt3, Wnt3A, Wnt8, Wnt8B, and Wnt10B (Wnt1 shares 96% identity in amino acid sequence to Wnt10B and overlap in function (51)) and induce secondary body axis in *Xenopus* and activates the canonical Wnt/β-catenin pathway. The Wnt5A class cannot induce secondary body axis formation in *Xenopus* and includes Wnt4, Wnt5A, Wnt5B, Wnt6, Wnt7A, and Wnt11 (see Ref. 52 for a review). Studies in zebrafish embryos indicate that Wnt5A and Wnt11 but not Wnt8 overexpression induce calcium release (11). However, little is known about the ability of Wnts, other than Wnt5A, to induce [Ca^2+^]*_i_* release in prostate or other mammalian cells.

However, these distinctions have been blurred when it was shown that Wnts associated with the so-called non-canonical pathway are capable of transforming cells in culture (13, 53, 54). In addition, the ligands of Wnt family are likely to share the cell membrane receptor, and most cells are exposed to a variety of Wnt ligands simultaneously. A balance of these signals and the pathways they might activate (*e.g.* Wnt/β-catenin or Wnt/Ca^2+^ or both) could determine, for example, cell fate of a stem cell or whether cancer cells metastasize (*e.g.* by acquiring increased capacity for motility). Wnt 11 is the only ligand tested in our study that did not mobilize [Ca^2+^]*_i_* (supplemental Movie SM8). A possible explanation for this may reside in primary amino acids that deviates from the consensus Wnt sequence in five amino acid residues, in close proximity to RWNC motif (supplemental Fig. S15).

##### β-Catenin Translocation into Nucleus Subsequent to Increase Nucleoplasmic Ca^2+^ and Depolarization of the NE

Macromolecular transport across the NE is comprised of a variety of independent and interdependent biochemical and electrochemical processes (23, 24). These may involve a nuclear localization signal binding of proteins intended for the nucleus with other cytosolic proteins to facilitate their entry requiring energy-dependent mechanisms or phosphorylation of specific proteins (55). Although β-catenin does not contain nuclear localization signal, by using *in vitro*, biochemical experiments, Sharma and colleagues (56) have shown that this recognition may occur by the interaction of the Arm domain of β-catenin with NPC component proteins. Upon reaching the outer NE in response Wnt signaling, β-catenin faces considerable barriers to entry into the nucleoplasm. Sharma *et al.* (56) describe the targeting of β-catenin to the outer NE, but their experiments do not explain how β-catenin may overcome significant biophysical hurdles (a molecular mass of 92 kDa and an overall negative charge of −20.8 at pH 7.0) to traverse the NE.

Other mechanisms involve silencing of NPC channel activity for transport of medium-sized (∼40 kDa) transcription factors (such as NFκB or SP1) (57). Ca^2+^ ions also play a role in the trans-NE transport of macromolecules (31), and it has been proposed that depletion of intranuclear Ca^2+^ can inhibit macromolecular transport of particularly intermediate size (70 kDa) but also small (<10 kDa) macromolecules across NE (25). Translocation of β-catenin, a 92-kDa transcription factor, from the cytosol to the nucleus is a key event in the so-called canonical Wnt signaling; however, the mechanism of this translocation is not known. The increase in intranuclear Ca^2+^ and depolarization of the nuclear membrane (Fig. 5 and supplemental Fig. S8) and reduction in β-catenin translocation into the nucleus in the presence of thapsigargin (supplemental Fig. S11) suggest a putative role of membrane potential regulating ions as regulators of Wnt signaling in PC3 cells.

In the convergent model of Wnt signaling in mammalian cells proposed here (Fig. 8), binding of a variety of Wnt ligands to their receptors activates [Ca^2+^]*_i_* release (Fig. 1). The sharp and transient increase in [Ca^2+^]*_i_* results in the increased intranuclear Ca^2+^ within 300 s of Wnt ligand activation (Fig. 5). Subsequent to intranuclear Ca^2+^ increase and depolarization of the NE, there is also an evident increase in the nuclear expression of β-catenin (Figs. 6 and 7). The model presented here does not exclude upstream homing events that may direct β-catenin to the NE or downstream events such as involvement of energy expenditure or other biochemical and macromolecular transport mechanisms that may also facilitate the ultimate translocation of β-catenin into the nucleus. It, however, includes an important and as yet unconsidered electrochemical aspect in the transport of β-catenin into the nucleus where its functional activity lies. It also suggests, at least in mammalian cell lines, an integrated signaling process that can facilitate, amplify and regulate activities of multiple transducers activated by individual ligands (Fig. 8).

## Footnotes

2 The abbreviations used are:

NE: nuclear envelope
DT: dwell time
NPC: nuclear pore complex.
